# Oncoproteomic Analysis Reveals Co-Upregulation of RELA and STAT5 in Carboplatin Resistant Ovarian Carcinoma

**DOI:** 10.1371/journal.pone.0011198

**Published:** 2010-06-18

**Authors:** Natini Jinawath, Chanont Vasoontara, Artit Jinawath, Xueping Fang, Kejia Zhao, Kai-Lee Yap, Tong Guo, Cheng S. Lee, Weijie Wang, Brian M. Balgley, Ben Davidson, Tian-Li Wang, Ie-Ming Shih

**Affiliations:** 1 Departments of Pathology, Oncology, and Gynecology and Obstetrics, Johns Hopkins Medical Institutions, Baltimore, Maryland, United States of America; 2 Research Center, Faculty of Medicine Ramathibodi Hospital, Mahidol University, Bangkok, Thailand; 3 Department of Pathology, Faculty of Medicine Ramathibodi Hospital, Mahidol University, Bangkok, Thailand; 4 Department of Chemistry and Biochemistry, University of Maryland, College Park, Maryland, United States of America; 5 Human Genome Sciences, Rockville, Maryland, United States of America; 6 Calibrant Biosystems, Gaithersburg, Maryland, United States of America; 7 Department of Pathology, Norwegian Radium Hospital, Oslo University Hospital, University of Oslo, Oslo, Norway; 8 Medical Faculty, University of Oslo, Oslo, Norway; Dresden University of Technology, Germany

## Abstract

**Background:**

Ovarian cancer is one of the most lethal types of female malignancy. Although most patients are initially responsive to platinum-based chemotherapy, almost all develop recurrent chemoresistant tumors and succumb to their diseases. Elucidating the pathogenesis underlying drug resistance is fundamental to the development of new therapeutics, leading to improved clinical outcomes in these patients.

**Methods and Findings:**

We compared the proteomes of paired primary and recurrent post-chemotherapy ovarian high-grade serous carcinomas from nine ovarian cancer patients using CIEF/Nano-RPLC coupled with ESI-Tandem MS. As compared to their primary tumors, more than half of the recurrent tumors expressed higher levels of several proteins including CP, FN1, SYK, CD97, AIF1, WNK1, SERPINA3, APOD, URP2, STAT5B and RELA (NF-κB p65), which were also validated by quantitative RT-PCR. Based on shRNA screening for the upregulated genes in *in vitro* carboplatin-resistant cells, we found that simultaneous knockdown of RELA and STAT5B was most effective in sensitizing tumor cells for carboplatin treatment. Similarly, the NF-κB inhibitor, BMS-345541, and the STAT5 inhibitor, Dasatinib, significantly enhanced cell sensitivity to carboplatin. Moreover, both RELA and STAT5 are known to bind to the promoter region of Bcl-X, regulating its promoter activity. In this regard, augmented Bcl-xL expression was detected in carboplatin-resistant cells. Combined ectopic expression of RELA and STAT5B enhanced Bcl-xL promoter activity while treatment with BMS-345541 and Dasatinib decreased it. Chromatin immunoprecipitation of the Bcl-X promoter region using a STAT5 antibody showed induction of RELA and STAT5 DNA-binding segments both in naïve cells treated with a high concentration of carboplatin as well as in carboplatin-resistant cells.

**Conclusions:**

Proteomic analysis identified RELA and STAT5 as two major proteins associated with carboplatin resistance in ovarian tumors. Our results further showed that NF-κB and STAT5 inhibitor could sensitize carboplatin-resistant cells and suggest that such inhibitors can be used to benefit patients with carboplatin-resistant recurrent ovarian cancer.

## Introduction

Ovarian cancer is the most lethal gynecological malignancy in the United States with an estimated 21,550 new cases and 14,600 deaths in 2009 [1]. Among all histological types of ovarian epithelial carcinoma, high-grade serous carcinoma is the most common and aggressive type, and is referred to generally as “ovarian cancer”. High-grade serous carcinoma is highly malignant with a 5-year survival rate of less than 30%. The majority of patients are diagnosed late after tumor cells have disseminated within the peritoneal cavity when surgical and medical intervention is far less effective. Patients with advanced stage disease are treated with cytoreduction surgery followed by carboplatin-based chemotherapy. Despite initial responsiveness to combined carboplatin and paclitaxel chemotherapy, most patients develop chemoresistant tumors and ultimately succumb to the recurrent disease [2]. Thus, elucidating the pathogenesis of chemoresistance is fundamental to the development of new therapeutics to overcome drug resistance in ovarian cancer patients.

To elucidate the molecular mechanisms of drug resistance investigators have employed several genome-wide techniques, including transcriptome analysis, to identify genes and their associated pathways in developing chemoresistance. As a result, a number of new drug resistant-associated genes have been identified. For example, we have reported that the expression levels of Nac1, Rsf-1 (HBXAP), fatty acid synthase and annexin A11 were significantly higher in recurrent, high-grade ovarian serous carcinoma specimens after chemotherapy and, more importantly, expression of these genes played a causal role in conferring drug resistance *in vitro* [3], [4], [5], [6], [7], [8]. In addition to genomic approaches, comparison of the proteomes between primary and recurrent post-chemotherapeutic carcinomas could represent another effective approach to identifying proteins involved in developing drug resistance. Proteins are the ultimate functional products of genetic information, and the application of proteomics approaches such as 2D difference gel electrophoresis, MALDI imaging mass spectrometry, electron transfer dissociation mass spectrometry and reverse-phase protein array analysis has identified novel disease-related proteins in cancer [9], [10].

In our effort to elucidate the pathogenesis of drug resistance in ovarian cancer, we have employed oncoproteomics as a discovery tool to identify the proteins preferentially expressed in recurrent post-chemotherapy ovarian carcinoma tissues using capillary isoelectric focusing (CIEF) coupled with nano-reversed-phase liquid chromatography (RPLC) for concentrating and resolving intact proteins. This approach has been shown to enhance the capacity to analyze complex protein mixtures from a limited amount of a specimen [11]. Candidate proteins were screened for their contribution to carboplatin resistance using shRNAs and *in vitro* chemoresistant cell models. In the results reported here, we show that RELA (p65 subunit of NF-κB) and STAT5 are the major proteins associated with carboplatin resistance in ovarian cancer, and show that they both bind the promoter and act synergistically to induce the expression of *Bcl-xL*, the molecular convergence point of the NF-κB and STAT5 pathways. Co-inhibition of these two pathways by RNAi or small compound inhibitors represents an attractive strategy for a combined therapeutic intervention of recurrent carboplatin-resistant ovarian cancer.

## Materials and Methods

### Ethics Statement

Acquisition of tissue specimens and clinical information were approved by an institutional review board (Johns Hopkins Medical Institutions) and by the Regional Ethics Committee (Norwegian Radium Hospital). The written informed consents were obtained from all patients.

### Clinical Samples and Cell lines

Ovarian cancer effusion samples were obtained from the Norwegian Radium Hospital. These included 9 pairs of matched primary and recurrent high-grade ovarian serous carcinoma ascites cell pellets (Table S1). Human cancer cell lines including 293T, OVCAR3, SKOV3, and A2780 cells were originally purchased from ATCC (Rockville, MD) and were cultured in RPMI1640 medium supplemented with 5% fetal bovine serum. To generate *in vitro* chemoresistant ovarian cancer cells, SKOV3, A2780, and OVCAR3 cells were selected by sustained treatment with different concentrations of carboplatin ranging from 0.2 µg/ml–4.0 µg/ml. Resistant colonies of each cell line were pooled and used for subsequent experiments. Chemoresistant cell lines were maintained in selective medium containing the highest tolerable concentrations of carboplatin (2.0 µg/ml for SKOV3-CR, 1.0 µg/ml for A2780-CR, and 0.5 µg/ml for OVCAR3-CR) [6]. Figures S1 A and S1 B show drug sensitivity assays (carboplatin IC_50_) and growth curve analysis of A2780-CR, SKOV3-CR and OVCAR3-CR as compared to their naïve counterparts.

### Proteomics analysis

Proteins extracted from clinical samples were analyzed using integrated Capillary Isoelectric Focusing (CIEF)/Nano-Reversed Phase Liquid Chromatography (Nano-RPLC) Separations Coupled with ESI-Tandem MS (Electrospray Ionization -Tandem Mass Spectrometry). The Open Mass Spectrometry Search Algorithm (OMSSA) developed at the National Center for Biotechnology Information was used to search the peak list files against a decoyed SwissProt human database. A total of 6,711 proteins were identified at a 1% false discovery rate (FDR) as determined by the target-decoy search method (see supplemental materials: Text S1, Figures S2 and S3 for detailed explanation). The 24 candidate proteins with high peptide counts and low paired t-test p-values that exhibited more than 2-fold upregulation in at least 4 out of 9 recurrent cases compared to their primaries were selected for subsequent validation by quantitative RT-PCR. The expression data of all proteins identified in this study are shown in Table S2.

### Drug sensitivity and cell viability assays

The shRNA plasmids targeting candidate genes and the control plasmid (pLKO.1-puro vector only) were obtained from Sigma. The shRNA nucleotide sequences are listed in Table S3. Transfection was performed using the Fugene HD (Roche) (293T, A2780, SKOV3-CR, A2780-CR), and the Nucleofection kit V (program V-05 for SKOV3) (Amaxa Biosystems). For each gene, shRNAs that showed the best knockdown effect among the 4 or 5 shRNAs screened were subsequently selected for drug sensitivity experiments. Table S4 shows the knockdown efficiency of all shRNAs in SKOV3-CR cells using qRT-PCR. For drug sensitivity assays, cells were transfected with targeting and control shRNAs, selected with Puromycin for 5–7 days, and then seeded into 384-well plates at a density of 750 cells/well. After overnight culture, the cells were treated with a series of concentrations of carboplatin and/or BMS-345541 (Cat.401480, Calbiochem), and/or Dasatinib (D-3307, LC Laboratories). Four days after transfection (i.e., three days after drug treatment), viable cells were counted by CellTiter-Blue™ (Promega) using a fluorescence microplate reader (Fluostar from BMG, Durham, NC). Data were determined from three replicates and are expressed as the percentage of control group without carboplatin treatment.

### Quantitative real-time reverse transcription-PCR

An RNA extraction kit (Qiagen) was used to extract total RNA, and the Superscript II First-strand cDNA synthesis kit (Invitrogen) was used to generate cDNA. Real-time PCR was performed on a Bio-Rad iCyclers (MyIQ, IQ4), and data analysis was performed using the Bio-Rad IQ5 v2 software. The PCR primers used in this study are listed in Table S5. The mean Ct of the gene of interest was calculated from replicate measurements and normalized with the mean Ct of a control gene, GAPDH, for which expression was relatively constant.

### Western blot

Western blot analysis was conducted using a standard protocol. Mouse monoclonal antibodies that react to Stat5b (G-2) (sc-1656, Santa Cruz Biotechnology) and the active subunit (12H11) of RELA (NF-κB p65) (MAB3026, Millipore) were used at a 1∶400 dilution, and a rabbit anti-GAPDH polyclonal antibody (Sigma, Cat # G9545) was used at a 1∶4,000 dilution. Similar amounts of total protein from each lysate were loaded and separated on 4–12% Tris-Glycine-SDS polyacrylamide gels (Novex, San Diego, CA) and electroblotted to Millipore Immobilon-P polyvinylidene difluoride membranes. Western blots were developed by chemiluminescence (Pierce, Rockford, IL).

### Apoptosis assay

Cells were treated with DMSO, and/or carboplatin (6 µg/ml), and/or BMS-345541 (5 µM), and/or Dasatinib (100 nM) for 48 hours, and then analyzed using an Annexin V-FITC apoptosis detection kit (BioVision) according to the manufacturer's protocol. The apoptotic population (Annexin V-positive) was measured by Flow Cytometry (Ex = 488 nm; Em = 530 nm) using FITC signal detector (FL1), and the phycoerythrin emission signal detector (FL2) for PI-positive cells.

### Luciferase reporter assay and CAT ELISA assay

For luciferase assay, cells were transfected with the pGL2 firefly reporter vector (Promega) or with either pGL2-Stat5-wt or pGL2-Stat5-mut containing a 1.0 kb Bcl-X promoter without or with, respectively, a point mutation in the Stat5 consensus binding sequence (C/T) (a generous gift from Dr Fabrice Gouilleux) [12]. Human pCMV-RELA and pCMV-HA were kind gifts from Dr. Paul J Chiao [13] and pCMV-STAT5B was a kind gift from Dr. Amy H. Bouton [14]. pRL-Renilla reporter vector (Promega), which serves to monitor transfection efficiency, was co-transfected with the pGL2 plasmid. Luciferase activity was determined using Dual-Glo luciferase reagent (Promega). Firefly luciferase activity was normalized to luminescence from Renilla luciferase and the ratio of luminescence from the experimental reporter to luminescence from the control reporter was calculated. For CAT assay, cells were co-transfected with pRL-Renilla and various CAT reporter constructs in pCAT-Basic (Promega). These were: −298/+22 wt, −298/+22κb mut, −207/+22, and−137/+22, which were a generous gift from Dr. Celine Gelinas [15]. The −298/+22κb mut construct has three point mutations in the NF-κB p65 consensus binding site in the Bcl-X promoter (GGG/TTT). Forty-eight hours after transfection, CAT assays were performed using a CAT ELISA assay kit (Roche) and Renilla luminescence served as a transfection efficiency control.

### Chromatin immunoprecipitation (ChIP) assay

Chromatin immunoprecipitation (ChIP) was performed using the ChIP-IT enzymatic kit (Active motif) on untreated carboplatin-resistant cells and their naïve counterparts, as well as on naïve cells treated with carboplatin (12 µg/ml) for 24 h prior to fixation with 1% paraformaldehyde. The lysates of fixed cells were treated with an enzymatic shearing cocktail (Active Motif, Carlsbad, CA) and the soluble fraction was incubated with 4 µL of anti-Stat5 (C-17) (sc-835X, Santa Cruz Biotechnology) or normal rabbit IgG, and precipitated with protein-G magnetic beads at 4°C overnight. To reverse crosslinking immunoprecipitated DNA was heated at 65°C for 6 hours, followed by proteinase K (10 µg/ml) treatment. The detection primers for the Bcl-X promoter included −824 bp to −588 bp (5′ side of RELA and STAT5 binding site), −412 bp to −330 bp (contains RELA and STAT5 sites), and −283 bp to −117 bp (3′ side). Quantitative PCR was performed using the SYBR Green-based detection system as described above with PCR primers that amplified different Bcl-X promoter regions (primer sequences are shown in Table S5). Input DNA was defined as an aliquot of sheared chromatin prior to immunoprecipitation, and was used to normalize the amount of chromatin used in each experiment.

## Results

### Identification and validation of candidate proteins in recurrent ovarian serous carcinoma

**Figure 1:**
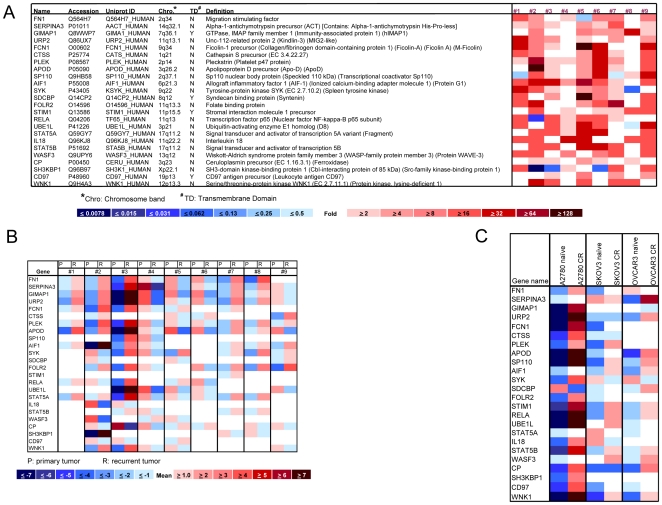
Identification and validation of candidate proteins in recurrent ovarian serous carcinoma. A) Proteomics analysis showing the expression ratio of recurrent to primary tumor in 24 candidate proteins. Nine pairs of matched primary and recurrent serous carcinomas (from #1 to #9) were analyzed and the pseudo-color codes represent the fold change (ratio of recurrent to primary tumor) from low (blue) to high (red). B) mRNA expression of candidate genes in recurrent and primary tumors using the same 9 pairs of samples utilized in proteomics analysis. Color codes represent the relative expression levels from low (blue) to high (red). C) mRNA expression of candidate genes in chemoresistant ovarian cancer cells; CR  =  Carboplatin Resistant. Color codes represent the relative expression levels from low (blue) to high (red).

The cell pellets from 18 ascites samples including the paired primary and recurrent post-chemotherapy ovarian high-grade serous carcinomas (Table S1) were analyzed using capillary isoelectric focusing/nano-reversed phase liquid chromatography coupled with ESI-tandem MS. Levels of 24 proteins were more than 2 fold higher in recurrent tumor cells compared to matched primary tumor cells from the same patient in at least 4 pairs of samples (Figure 1A). Candidate proteins upregulated in recurrent tumors included cell adhesion proteins, cell-extracellular matrix interaction proteins, cell surface glycoproteins (FN1, STIM1, CD97), protein kinases (SYK, WNK1), acute phase proteins (SERPINA3, CTSS), proteins induced by pro-inflammatory cytokines, interferon and growth factors (AIF1, RELA, IL18, STAT5A, STAT5B). The expression of the 24 candidate proteins was validated using quantitative RT-PCR in the same set of clinical samples, as well as in the three pairs of carboplatin-naïve and resistant (CR) ovarian cancer cell lines (Figure 1B and 1C). We found that the majority of the 24 candidate genes had higher mRNA expression levels in recurrent tumors than in primary tumors (Figure 1B), and that the carboplatin-resistant cells also had higher mRNA levels than naïve cells (Figure 1C).

### Identification of RELA and STAT5B as the major carboplatin-resistant proteins in recurrent ovarian serous carcinoma

**Figure 2:**
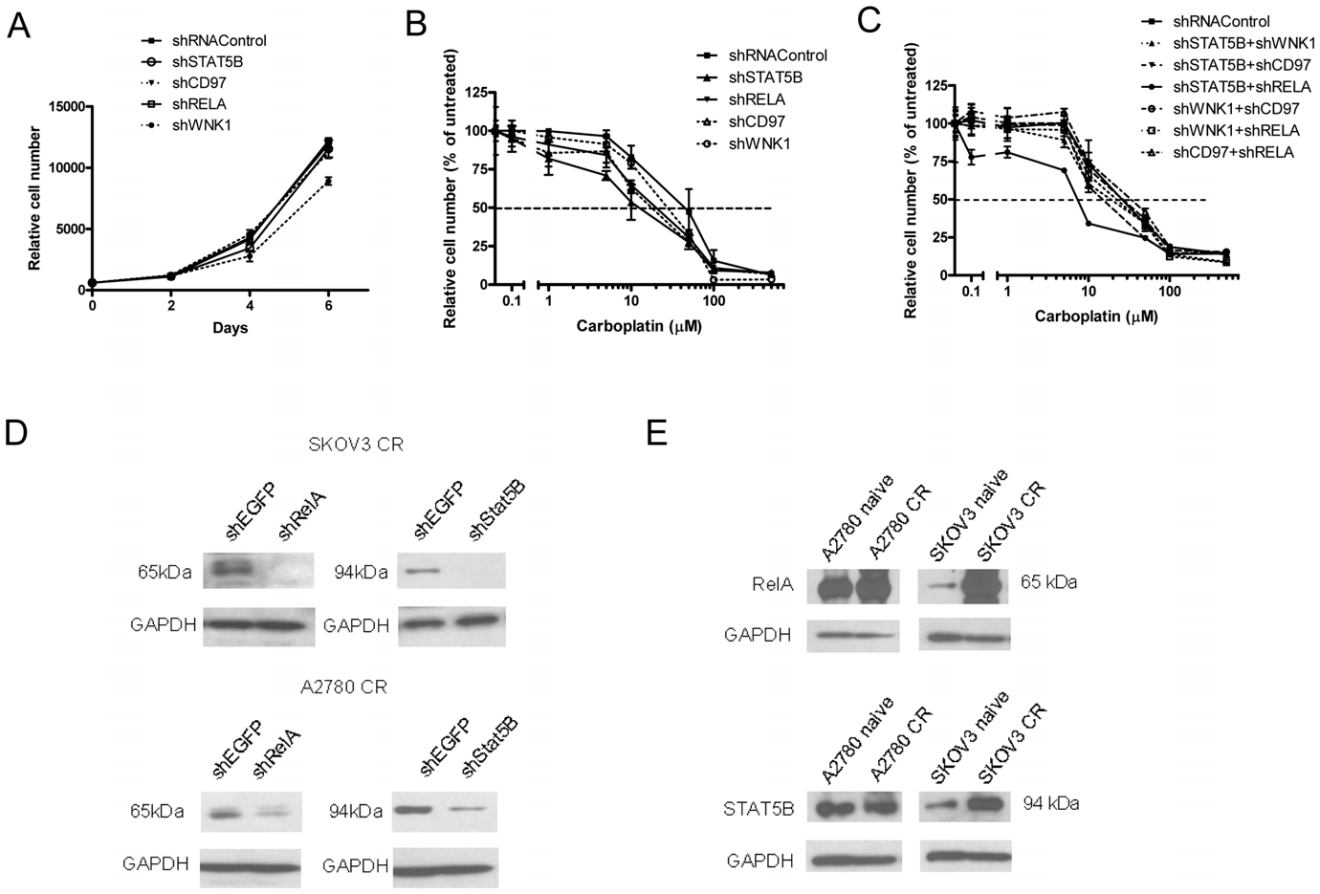
Identification of RELA and STAT5B as the major carboplatin-resistant genes in recurrent ovarian serous carcinoma. A) SKOV3 carboplatin-resistant cells (SKOV3-CR) were transfected with candidate gene-specific shRNAs, and cell growth was monitored for 6 days. B) SKOV3-CR cells were transfected with specific shRNAs against each candidate gene (STAT5B, WNK1, RELA, CD97), and selected with puromycin for 5–7 days. The cells were then treated with various concentrations of carboplatin for 4 days, and the IC_50_ was determined for each gene by cell viability assay. C) SKOV3-CR cells were co-transfected with combinations of two shRNAs against STAT5B, WNK1, RELA, CD97, and selected with puromycin for 5–7 days. The cells were then treated with various concentrations of carboplatin for 4 days, and IC_50_ was determined for each gene by cell viability assay. D) Western blots showing reduced RELA and STAT5B expression 48 hours after transfection of their specific shRNAs in SKOV3-CR and A2780-CR cells. E) Western blots showing endogenous expression level of RELA and STAT5B in A2780 naïve, A2780-CR, SKOV3 naïve, and SKOV3-CR cells.

In order to prioritize the recurrent tumor-associated candidate proteins for assessment of their roles in chemoresistance *in vitro*, we selected 11 genes validated by RT-PCR including *CP, STAT5B, FN1, CD97, RELA (NF-κB p65), AIF1, SYK, WNK1, URP2, SERPINA3* and *APOD* because they were upregulated in at least 2 out of 3 pairs of carboplatin-resistant cells. We used shRNAs to knockdown their expression in SKOV3-CR cells. Growth curve analyses were carried out to determine whether shRNA knockdown of the candidate genes had an effect on cell proliferation in the absence or presence of carboplatin (see Figure S4 for all screening results). Among the 11 genes, suppression of *STAT5B, RELA*, *CD97*,and *WNK1* had a minimal effect on cell proliferation (Figure 2A), but significantly sensitized chemoresistant cells to carboplatin (Figure 2B). The IC_50_ of STAT5B, CD97, RELA, and WNK1 were 12.5 µM, 17.8 µM, 19.8 µM, and 27.3 µM, respectively, compared to 44.2 µM by the control shRNA. Because drug resistance is likely to involve several genes and pathways, we simultaneously knocked down the expression of two of these genes, and found that combined suppression of *STAT5B* and *RELA* was most effective, as the IC_50_ decreased to 7.3 µM as compared to 26.6 µM by control shRNA under this experimental condition (Figure 2C). Figure 2D shows the protein knockdown effect by the specific shRNAs in both SKOV3-CR and A2780-CR cell lines. Moreover, Western blot using specific antibodies against activated RELA and STAT5B demonstrated higher protein expression levels in SKOV3-CR cells and to a lesser extent in A2780-CR cells prior to knockdown as compared to their naïve counterparts (Figure 2E).

### Co-expression of RELA and STAT5 in chemoresistant cells upregulated *Bcl-xL* transcription

**Figure 3:**
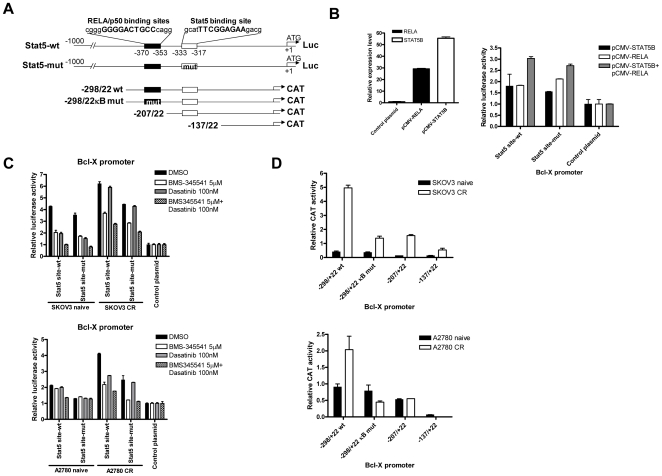
Co-induction of RELA and STAT5 in chemoresistant cells results in upregulated Bcl-xL transcription. A) Schematic illustration of the six Bcl-X reporter constructs used in this study. The name of CAT constructs reflects their length relative to the first nucleotide in the *Bcl-xL* cDNA. STAT5-mut contains a point mutation in the Stat5 consensus binding site, while -298/22κB mut has three base substitutions in the RELA/p50 consensus binding site. B) Bcl-X promoter assay using 293T cells transfected with STAT5-wt or STAT5-mut or control reporter plasmid, plus pCMV-RELA and/or pCMV-STAT5B. Luciferase activity was measured 24 hours after transfection (right panel); exogenous *RELA* and *STAT5B* expression level at 24 hours after transfection of pCMV-RELA or pCMV-STAT5B or control plasmid (left panel). C) Bcl-X reporter assay in chemoresistant cells and their naïve counterparts transfected with STAT5-wt or STAT5-mut or control reporter plasmid (top panel: SKOV3; bottom panel: A2780). Luciferase activity was measured 24 hours after treatment with 5 µM BMS-345541 and/or 100 nM dasatinib and/or DMSO. D) CAT ELISA assay of chemoresistant cells and their naïve counterparts. CAT activity was measured 48 hours after transfection with the CAT-reporter plasmids (top panel: SKOV3; bottom panel: A2780)

RELA and STAT5 proteins have specific binding sites on the Bcl-X promoter, and regulate one of the two *Bcl-X* isoforms, *Bcl-xL*, which is an anti-apoptotic protein involved in maintaining cell survival under stresses such as genotoxic drugs. Thus, it is likely that RELA and STAT5 proteins cooperate in inducing Bcl-X promoter activity and synergistically enhance *Bcl-xL* expression in chemoresistant ovarian cancer cells. To test this hypothesis, we performed reporter assays and chromatin immunoprecipitation in 293T and ovarian cancer cell lines. Figure 3A illustrates the five reporter constructs used in the analyses. Specifically, the STAT5-mut harbored a point mutation in the STAT5 consensus binding site, while the −298/22κB mut had three base substitutions in the RELA/p50 consensus binding site. In Figure 3B, the Bcl-X promoter assay using STAT5 wt and STAT5 mut constructs showed the highest reporter activity in 293T cells after introducing exogenous expression of both RELA and STAT5B. Interestingly, although the point mutation in STAT5 binding site was shown to completely eliminate binding activity in Ba/F3 murine pro-B cells [12], it revealed only a subtle decrease in the Bcl-X promoter activity as shown here. We next investigated regulation of the Bcl-X promoter by endogenous RELA and STAT5 expression in SKOV3-CR, A2780-CR and their naïve counterparts. Cells treated with a combination of 5 µM BMS-345541, a highly-selective IkB kinase inhibitor [16], and 100 nM dasatinib, a protein-tyrosine kinase inhibitor that inhibits STAT5 signaling [17], demonstrated the lowest Bcl-X reporter activity of STAT5 wt and STAT5 mut constructs, followed by BMS-345541 alone, dasatinib alone, and DMSO alone (Figure 3C). Both carboplatin-resistant cells exhibited higher Bcl-X promoter activity than their naïve counterparts. Reduced *Bcl-xL* expression levels after treatment with 5 µM BMS-345541 or 100 nM dasatinib for 24 hours were also confirmed in both SKOV3-CR and A2780-CR cells (data not shown). As shown in Figure 3D, cells transfected with the −298/22κB wt construct, which had both wild-type binding sites of RELA/p50 and STAT5, had the highest reporter activity, followed by −298/22κB mut and −207/+22 constructs, which contained only one intact STAT5 binding site. The −137/+22 construct, depleted of both binding sites, showed the lowest activity. These observations indicated that carboplatin-resistant cells had higher Bcl-X reporter activity than their naïve counterparts.

**Figure 4:**
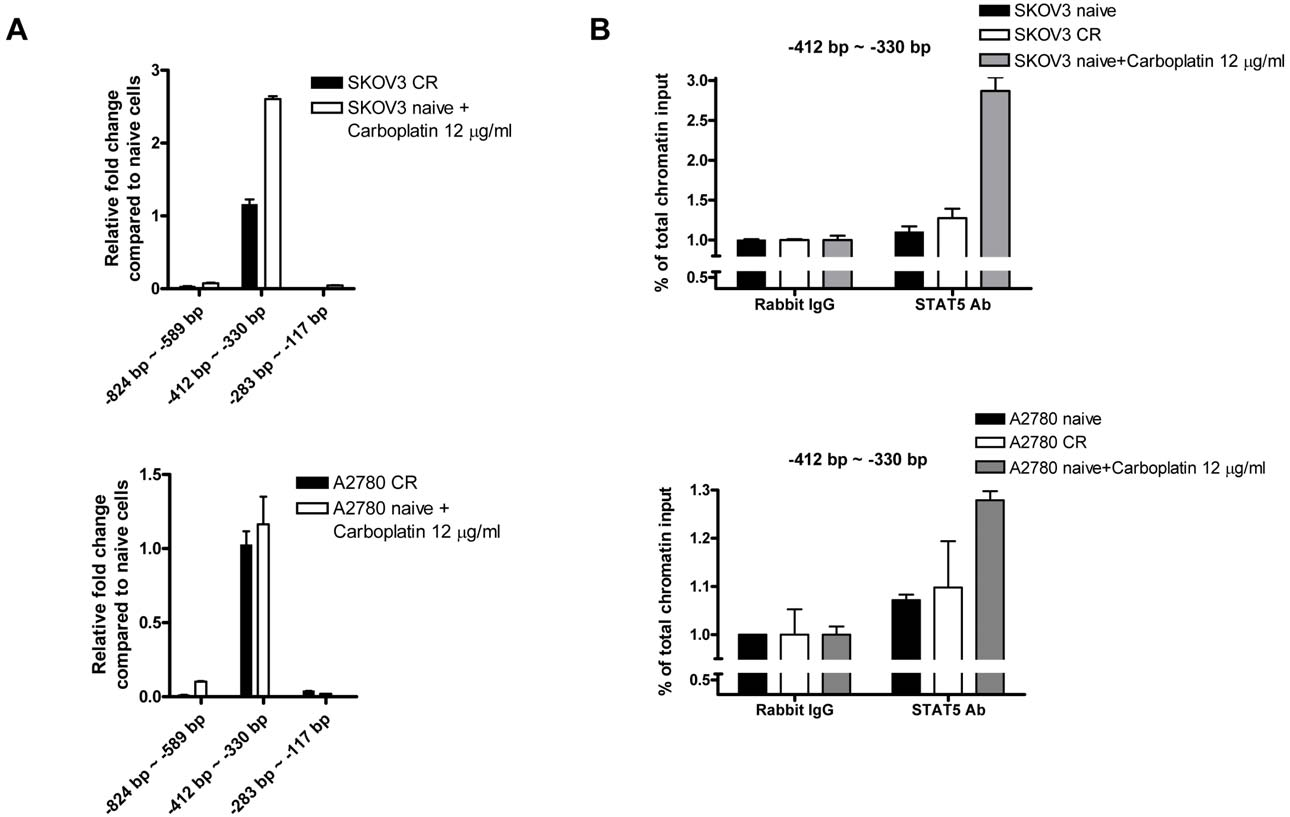
Chromatin immunoprecipitation (ChIP) assay of the Bcl-X promoter using STAT5 antibody in chemoresistant cells, their naïve counterparts, and naïve cells treated with high-dose carboplatin. A) Relative fold enrichment of immunoprecipitated genomic fragments by quantitative PCR in chemoresistant cells and in naïve cells treated with 12 µg/ml carboplatin for 24 hours, as compared to the untreated naive counterparts. Fragment “−412 bp∼−330 bp” contains both RELA and STAT5 binding sites, while fragment “−824 bp∼−588 bp” on the 5′ side of RELA and STAT5 binding sites and fragment “−283 bp∼−117 bp” on the 3′ side serve as negative controls. Top panel: SKOV3; bottom panel: A2780. B) STAT5-bound chromatin in relation to % of total chromatin input of the “−412 bp∼−330 bp” region. Rabbit IgG-bound chromatin serves as control. Top panel: SKOV3; bottom panel: A2780.

We performed chromatin immunoprecipitation (ChIP) analysis using an antibody against STAT5 and quantified the pulled down chromatin fragments using quantitative PCR. We used untreated SKOV3-CR, A2780-CR, and their naïve counterparts, as well as naïve cells treated with a high dose carboplatin (12 µg/ml) as a positive control. The results in Figures 4A and 4B show that in a resting state, chemoresistant cells exhibited a subtle increase in STAT5-DNA binding to the Bcl-X promoter as compared to naïve cells, while naïve cells acutely treated with high dose carboplatin had the highest STAT5-DNA binding. Taken together, these results strongly suggest that RELA and STAT5 co-regulated Bcl-X promoter in response to carboplatin, and conferred chemoresistance in ovarian cancer, in part, through the transcriptional activation of *Bcl-xL*.

### *Bcl-xL* expression is enhanced in chemoresistant cells and its suppression reduces carboplatin resistance

**Figure 5:**
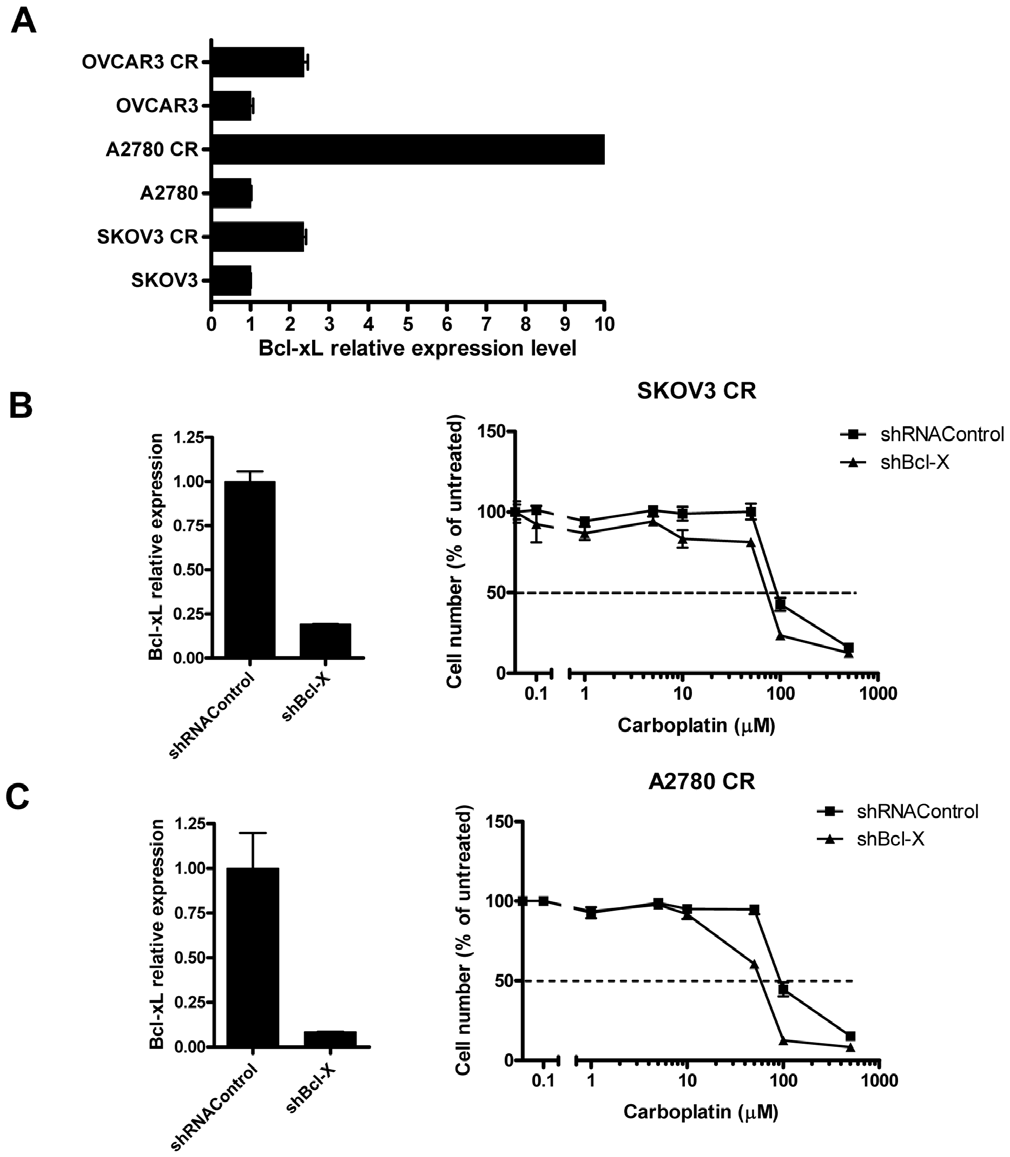
The expression level of Bcl-xL is increased in chemoresistant cells and its suppression reduces carboplatin resistance. A) mRNA expression of *Bcl-xL* in carboplatin-resistant and naïve ovarian cancer cells based on qRT-PCR. B and C) Knockdown effect of Bcl-xL shRNA in SKOV3-CR cells (B) and in A2780-CR cells (C). After transfection with Bcl-xL shRNA and selection with puromycin for 5–7 days, Bcl-xL expression level decreases (left panels). The cells were then treated with carboplatin at various concentrations for 4 days. IC_50_ curves were determined using cell viability assay (right panels).

To determine the anti-apoptotic effect of *Bcl-xL* upon carboplatin treatment, we first compared *Bcl-xL* gene expression levels in the three pairs of ovarian chemoresistant-naïve cell lines by quantitative RT-PCR and confirmed that *Bcl-xL* expression levels were higher in the chemoresistant cells as compared to their corresponding naïve cells, especially in A2780-CR (Figure 5A). Next, we knocked down *Bcl-xL* expression in SKOV3-CR and A2780-CR cells using the Bcl-xL-specific shRNA and determined their carboplatin IC_50_. As expected, reduced *Bcl-xL* expression resulted in moderate carboplatin sensitization in both cell lines (IC_50_ = 88.80 µM for shRNA control, 70.70 µM for Bcl-X shRNA (SKOV3-CR); 91.36 µM for shRNA control, 58.00 µM for Bcl-X shRNA (A2780-CR)) (Figures 5B and 5C).

### Co-inhibition of *RELA* and *STAT5B* expression by small molecule inhibitors synergistically sensitizes chemoresistant ovarian cancer cells to carboplatin

**Figure 6:**
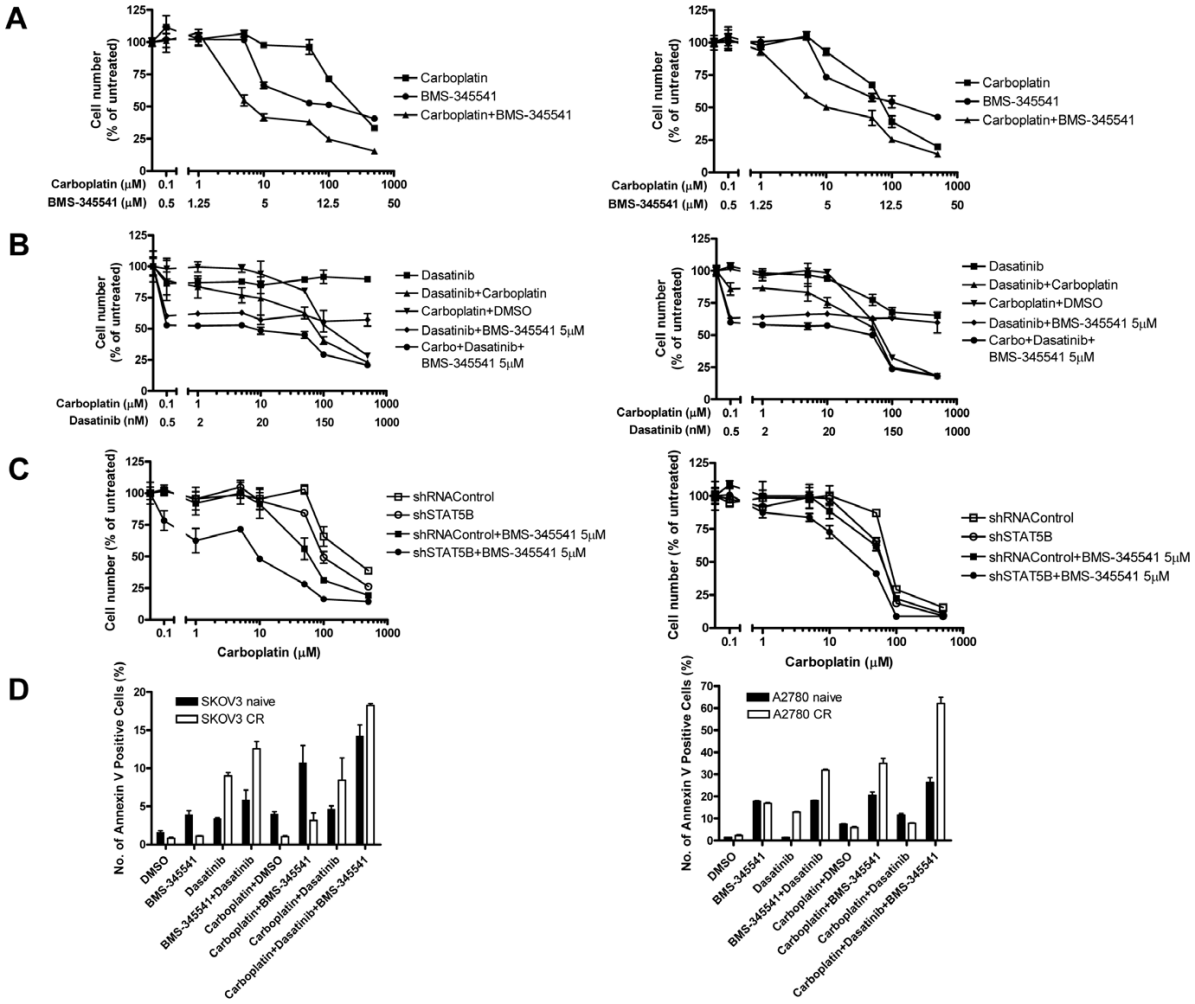
Co-inhibition of RELA and STAT5B pathways by small molecule inhibitors synergistically sensitizes chemoresistant ovarian cancer cells to cytotoxic effect of carboplatin. A) SKOV3-CR (left panel) and A2780-CR cells (right panel) were treated with a series of concentrations of carboplatin and BMS-345541 for 4 days before IC_50_ curves were obtained. B) SKOV3-CR (left panel) and A2780-CR cells (right panel) were treated with a series of dasatinib concentrations, or a series of carboplatin concentrations, or 5 µM BMS-345541 in combination with a series of dasatinib concentrations, or a series of concentrations of dasatinib and carboplatin, or 5 µM BMS-345541 in combination with a series of concentrations of dasatinib and carboplatin for 4 days before IC_50_ curves were measured. C) SKOV3-CR (left panel) and A2780-CR cells (right panel) were either transfected with STAT5B- or control shRNAs, with or without 5 µM BMS-345541 treatment, and subjected to a series of concentrations of carboplatin for 4 days before IC_50_ curves were determined. D) Annexin V assay to measure apoptosis of chemoresistant cells and their naïve counterparts after treatment with carboplatin (6 µg/ml) and/or BMS-345541 (5 µM) and/or dasatinib (100 nM) for 48 hours. Annexin V positive cells were quantified using flow cytometry. Left panel: SKOV3-CR; right panel: A2780-CR.

To extend the above findings that specific shRNAs against RELA and STAT5B effectively sensitized chemoresistant ovarian cancer cells to carboplatin killing, we sought to determine the effect of their small molecule inhibitors as potential chemosensitizers. SKOV3-CR and A2780-CR cells were cultured with various concentrations of carboplatin in combination with BMS-345541, a specific NF-κB signaling inhibitor. BMS-345541 markedly enhanced carboplatin-induced growth suppression (carboplatin IC_50_ = 6.27 µM for SKOV3-CR and 9.62 µM for A2780-CR) as compared to cells treated with BMS-345541 only (IC_50_ = 118.06 µM for SKOV3-CR and 181 µM for A2780-CR), and carboplatin only (IC_50_ = 247.61 µM for SKOV3-CR and 74.84 µM for A2780-CR) (Figure 6A). To determine if the STAT5 inhibitor, dasatinib (BMS-324825), could collaborate with BMS-345541 in sensitizing cell to growth inhibition by carboplatin, we treated the two carboplatin ovarian cancer cell lines with various concentrations of carboplatin and dasatinib and with a fixed concentration of BMS-345541 (5 µM, BMS-345541 IC_50_). IC_50_ analysis showed that chemoresistant cells treated with the combination of carboplatin, dasatinib, and 5 µM BMS-345541 exhibited the highest sensitization to carboplatin killing (IC_50_ = 7.03 µM for SKOV3-CR; 41.14 µM for A2780-CR), followed by those treated with dasatinib and carboplatin (IC_50_ = 74.84 µM for SKOV3-CR; 56.30 µM for A2780-CR), and carboplatin alone (IC_50_ = 125.00 µM for SKOV3-CR; 64.90 µM for A2780-CR) (Figure 6B). Of note, the growth of cells treated with various doses of dasatinib alone was not significantly reduced even at a concentration as high as 1000 nM.

As the chemosensitizing effects of dasatinib can be related to its ability to suppress several signaling pathways in addition to STAT5, we replaced dasatinib with STAT5B-specific shRNA in the IC_50_ assays to confirm that the effect of dasatinib observed was a result of STAT5 inhibition. We found that similar to dasatinib, STAT5B-specific shRNA synergized with BMS-345541 in conferring the best chemosensitization effect, again indicating that co-inhibition of STAT5 and NF-κB is a promising strategy for the treatment of carboplatin-resistant ovarian cancers (Figure 6C). To determine whether BMS-345541 and/or dasatinib exerted their carboplatin-sensitization effect via induction of carboplatin-induced apoptosis, we performed annexin V staining on chemoresistant cells and their naïve counterparts treated with 5 µM BMS-345541 and/or 100 nM dasatinib in combination with 6 µg/ml carboplatin. As shown in Figure 6D, cells treated with the combination of carboplatin plus low doses of BMS-345541 and dasatinib showed the highest percentage of annexin V-positive cells. Both SKOV3-CR and A2780-CR, and to a lesser extent their naïve counterparts, exhibited enhanced carboplatin-induced apoptosis when co-treated with BMS-345541 or dasatinib. In agreement with the earlier results, chemoresistant cells expressed higher amounts of RELA and STAT5B proteins than did the naïve cells, and may be more dependent on these pathways for cell survival, thereby becoming more sensitive to the inhibitors. It is also interesting to note that among the two chemoresistant cells used in our study, A2780-CR had a smaller induction of RELA and STAT5B expression in relation to its naïve counterpart as compared to SKOV3-CR (Figure 2E). Consistently, although A2780-CR was more sensitive to carboplatin treatment than SKOV3-CR, its response to the growth inhibitory effect of NF-κB and STAT5 inhibitors was to a lesser extent. This observation supports the notion that drug resistance is a complex phenotype and suggests that other molecular mechanisms may also participate in the development of carboplatin resistance in A2780-CR cells.

## Discussion

In this study, we used proteomic analysis and functional screening to identify RELA and STAT5B as the two major proteins involved in carboplatin resistance in ovarian cancer. We demonstrated that both proteins bound to the Bcl-X promoter and enhanced the expression of *Bcl-xL* which has been known to play a causal role in conferring carboplatin resistance in ovarian cancer through its anti-apoptotic functions [18]. Inhibition of either expression or activity of RELA and STAT5B decreased *Bcl-xL* expression levels and increased carboplatin sensitivity in carboplatin-resistant ovarian cancer cells. Our results provide new insight into the molecular processes underlying the development of carboplatin resistance in ovarian cancer and have important clinical implication.

The majority of the candidate proteins upregulated in recurrent tumors were proteins such as SERPINA3, AIF1, IL18, RELA, STAT5A and STAT5B that are either expressed in response to stimulation by pro-inflammatory cytokines or are involved in inflammatory networks. This observation is consistent with recent reports demonstrating that pro-inflammatory cytokines and inflammatory response pathways play an important role in ovarian cancer progression and chemoresistance [19], [20]. More specifically, Konstantinopoulos et al. have reported that NRF2, NF-κB, cytokines and proteins involved in inflammatory response pathways are highly induced by carboplatin treatment [19]. Similarly, one of the major findings in our study was the upregulation of RELA and STAT5B in recurrent post-chemotherapy ovarian cancer cells. RELA (NF-κB p65) is one of the three transcriptional activator units of NF-κB along with RELB and c-REL. The above findings raise the possibility that recurrent tumors are enriched by cells exhibiting a cancer stem cell-like phenotype. Ovarian cancer stem cell-like cells have been reported to exhibit the capacity to promote a pro-inflammatory microenvironment by constitutive NF-κB activity and cytokine and chemokine production. They are also proficient in DNA repair and are resistant to conventional chemotherapeutic agents [20]. Hence, in line with our findings, pro-inflammatory and cytokine regulatory proteins are likely major players in ovarian cancer chemoresistance either via the direct growth-promoting/anti-apoptotic effect or via the creation of growth-supportive niches/microenvironment for ovarian cancer stem cell-like cells.

Normally, the subcellular location of NF-κB is controlled by its binding to a family of inhibitory proteins, IκBs, which mask its nuclear localization signal, thus preventing nuclear localization. Phosphorylation of IκB by IκB kinase (IKK) leads to its ubiquitination and subsequent NF-κB translocation to the nucleus [21]. NF-κB is involved in the vast majority of stress-induced, immune, and inflammatory responses. It is also an important regulator in cell fate decisions, such as programmed cell death and proliferation control, and is critical in tumorigenesis. Its role in the development of chemoresistance in ovarian cancer has previously been reported [22], [23].

STAT5 isoforms including STAT5A and STAT5B mainly share a similar molecular structure and function. STAT5 has been demonstrated to be an essential molecule in the pathogenesis of leukemia [24] and solid tumors including liver cancer, lymphoma, head and neck cancer, and prostate cancer [25], [26], [27], [28]. Although our proteomics screening identified both STAT5A and STAT5B as upregulated proteins in recurrent ovarian cancer, we focused on studying STAT5B in our shRNA screens because it was also markedly upregulated in our chemoresistant cell lines. Interestingly, reduced STAT5B did not show growth suppression in chemoresistant cells, instead it sensitized the cells to carboplatin, suggesting a role in regulation of chemotherapy-induced apoptosis in ovarian cancer rather than direct participation in cellular proliferation.

In this study, we further demonstrated that, as compared to naïve cells, chemoresistant ovarian cancer cells showed increased levels of RELA and STAT5 binding to the Bcl-X promoter. The binding of both nuclear proteins to the Bcl-X promoter resulted in enhanced transcriptional activation of *Bcl-xL*. Moreover, we showed that co-inhibition of NF-κB and STAT5 pathways, either by specific shRNAs or small molecule inhibitors, synergistically reduced the chemoresistance potential in carboplatin-resistant ovarian cancer cells partly through suppression of *Bcl-xL* expression. Bcl-xL is known to be involved in ovarian cancer chemoresistance [29], [30], and the current study provides further evidence that *Bcl-xL* expression is significantly enhanced in carboplatin-resistant ovarian cancer cells. *Bcl-X* encodes two apoptosis regulators with opposing effects; Bcl-xL, a long form, exerts anti-apoptotic activity, while Bcl-xS, a short form, exerts proapoptotic activity. Although *Bcl-xL* and *Bcl-xS* share the same promoter, RELA/p50 [13], [15] and STAT5 [12], [31] have been shown to selectively induce the production of Bcl-xL in lymphoid cells. Our data demonstrate that as compared to naïve cells, chemoresistant ovarian cancer cells show increased RELA and STAT5-dependent transcriptional activation and protein-DNA binding at the Bcl-X promoter, supporting the view that carboplatin resistance in ovarian cancer cells is at least in part mediated by the NF-κB/STAT5-Bcl-xL axis.

Targeted therapy has shown promise in improving the clinical outcome in certain types of human cancer and it has been proposed that targeted strategies may be useful for enhancing the effects of chemotherapeutics. To this end, several small molecule inhibitors of the NF-κB pathway such as RTA-402 and Bortezomib, have been evaluated in clinical trials [32]. In the current study, we used dasatinib (BMS-324825), an FDA-approved inhibitor of Bcr-Abl and Src family kinases, to suppress the STAT5 pathway. Although a specific STAT5 inhibitor has not yet been developed, lestaurtinib (CEP701), a new JAK2 inhibitor that suppresses JAK2/STAT5 signaling is showing promise in clinical trials for myeloproliferative disorders and pancreatic cancer [33]. The recent emergence of clinically-tested inhibitors for both NF-κB and STAT5 pathways makes them promising candidates for targeted therapy of carboplatin-resistant tumors. Additionally, a small molecule inhibitor for Bcl-2/Bcl-xL is also available [18]. The capacity to target NF-κB, STAT5, and Bcl-2/Bcl-xL pathways simultaneously, and thus maximize the suppression of *Bcl-xL* expression, should be of great benefit to ovarian cancer patients who have developed carboplatin resistance.

In summary, we have compared the proteomes between primary and recurrent ovarian tumor samples and have identified several pro-inflammatory cytokines and nuclear proteins involved in the inflammatory response pathway in recurrent tumors. Among them, we found that preferential co-upregulation of NF-κB and STAT5 represents an important mechanism in developing carboplatin resistance in ovarian cancer. Furthermore, we showed that Bcl-xL is the converging molecule of the two pathways, and we provided evidence that one of the molecular mechanisms by which NF-κB and STAT5 upregulation contributes to carboplatin resistance is by preventing cell death through transcriptional activation of *Bcl-xL*. The findings presented here provide evidence of synergy between NF-κB and STAT5 in transcriptional activation of *Bcl-xL* in ovarian cancer cells, and also indicate potential molecular pathways to be targeted in order to enhance chemotherapeutic effects. Further molecular studies and future clinical trials using NF-κB and STAT5 inhibitors will address clinical benefits of targeting Bcl-xL as an approach toward enhancing the efficacy of chemotherapy in ovarian cancer patients.

## Supporting Information

Text S1 Supplemental materials and methods.
(0.04 MB DOC)

Figure S1 Carboplatin-resistant ovarian cancer cells A. Carboplatin IC_50_ of chemoresistant cells and their naïve counterparts [left panel: A2780; right panel: SKOV3]. B. Growth curve (cell viability assays) of carboplatin-resistant SKOV3, A2780, OVCAR3 cells and their naïve counterparts treated with 2 µg/ml, 1 µg/ml, and 0.5 µg/ml of carboplatin, respectively.
(0.60 MB TIF)

Figure S2 Paired t-test p-value distribution across the 6,711 proteins. A. A histogram plot of the p-value distribution across all proteins. B. A volcano plot of the p-value distribution across all proteins.
(0.59 MB TIF)

Figure S3 The distribution of case pairs showing more than 2-fold upregulation in recurrent compared to primary ovarian cancers.
(0.53 MB TIF)

Figure S4 All shRNAs screening results using SKOV3-CR cells. A. Growth curve analysis of SKOV3-CR transfected with each shRNA. B. Carboplatin IC_50_ of each shRNA screened.
(0.79 MB TIF)

Table S1 Clinical sample information.
(0.02 MB XLS)

Table S2 The expression of all proteins identified from the 9 pairs of primary and recurrent ovarian cancer samples (the 24 candidate proteins are highlighted in yellow).
(2.70 MB XLS)

Table S3 shRNA nucleotide sequences.
(0.02 MB XLS)

Table S4 Knock-down effect of specific shRNAs used in this study by qRT-PCR (SKOV3-CR).
(0.02 MB XLS)

Table S5 Primer sequences for qRT-PCR experiments.
(0.02 MB XLS)
