# Supplementary material for: Oncoproteomic Analysis Reveals Co-Upregulation of RELA and STAT5 in Carboplatin Resistant Ovarian Carcinoma
Source: PLoS One. 2010 Jun 18;5(6):e11198. doi: 10.1371/journal.pone.0011198 (PMC2887843; doi:10.1371/journal.pone.0011198)
Supplement: Text S1 — Supplemental materials and methods. (0.04 MB DOC) [file pone.0011198.s001.doc]

**Text S1: Supplementary materials and methods**

**Proteomics analysis**

**Materials and Reagents.** Fused-silica capillaries (50 μm i.d./375 μm o.d. and 100 μm i.d./375 μm o.d.) were acquired from Polymicro Technologies (Phoenix, AZ). Acetic acid, ammonium acetate, ammonium hydroxide, ampholyte 3-10, dithiothreitol (DTT), formic acid, and iodoacetamide (IAM) were obtained from Sigma (St. Louis, MO). Acetonitrile, sodium dodecyl sulfate (SDS), tris(hydroxymethyl)aminomethane (Tris), and urea were purchased from Fisher Scientific (Pittsburgh, PA). Sequencing grade trypsin was obtained from Promega (Madison, WI). All solutions were prepared using water purified by a Nanopure II system (Dubuque, IA) and further filtered with a 0.22 μm membrane (Millipore, Billerica, MA).

**Proteome Sample Preparation.** The isolated tumor cells were placed directly into a microcentrifuge tube containing 1% SDS and 20 mM Tris-HCl at pH 8.0. The soluble proteins were collected in the supernatant by centrifugation at 20,000 g for 30 min. The supernatant containing extracted proteins was placed in a dialysis cup and dialyzed overnight at 4 °C against 100 mM Tris-HCl at pH 8.2. The extracted and dialyzed proteins were reduced and alkylated by sequentially adding DTT and IAM with final concentrations of 10 mg/mL and 20 mg/mL, respectively. The solution was incubated at 37 ^0^C for 1 hr in the dark and then diluted 8-fold with 100 mM ammonium acetate at pH 8.0. Trypsin was added at a 1:40 (w/w) enzyme to substrate ratio and the solution was incubated at 37 ^0^C overnight. Tryptic digests were desalted using a Peptide MacroTrap column (Michrom Bioresources, Auburn, CA), lyophilized to dryness using a SpeedVac (Thermo, San Jose, CA), and then stored at -80 ^0^C.

**Integrated Capillary Isoelectric Focusing (CIEF)/Nano-Reversed Phase Liquid Chromatography (Nano-RPLC) Separations.** On-line integration of CIEF with nano-RPLC as a multidimensional peptide and protein separation platform has been described in detail in previous work^1-3^ and was employed for systematically resolving peptide digests based on their differences in isoelectric point (pI) and hydrophobicity. Briefly, an 80-cm long CIEF capillary (100 μm i.d./365 μm o.d.) was initially filled with a solution containing 2% ampholyte 3-10 and 1.5 mg/mL tryptic peptides. Peptide focusing was performed by applying electric field strength of 300 V/cm and using solutions of 0.1 M acetic acid and 0.5% ammonium hydroxide as the anolyte and the catholyte, respectively.

Focused peptides were sequentially fractionated by hydrodynamically loading into individual trap columns (3 cm x 200 μm i.d. x 365 μm o.d.) packed with 5 μm porous C_18_ reversed-phase particles. Each peptide fraction was subsequently analyzed by nano-RPLC equipped with an Ultimate dual-quaternary pump (Dionex, Sunnyvale, CA) and a dual nano-flow splitter connected to two pulled-tip fused-silica capillaries (50 μm i.d. x 365 μm o.d.). These two 15-cm long capillaries were packed with 3-μm Zorbax Stable Bond (Agilent, Palo Alto, CA) C_18_ particles.

Nano-RPLC separations were performed in parallel in which a dual-quaternary pump delivered two identical 2-hr organic solvent gradients with an offset of 1 hr. Peptides were eluted at a flow rate of 200 nL/min using a 5-45% linear acetonitrile gradient (containing 0.02% formic acid) over 100 min with the remaining 20 min for column regeneration and equilibration. Full scans were collected from 400 - 1400 m/z using a linear ion-trap mass spectrometer (LTQ, ThermoFinnigan, San Jose, CA) and 5 data dependent MS/MS scans were gathered with dynamic exclusion set to 18 sec.

**Data Analysis.** The Open Mass Spectrometry Search Algorithm (OMSSA) developed at the National Center for Biotechnology Information^4^ was used to search the peak list files against a decoyed SwissProt human database. This decoyed database was constructed by reversing all real sequences and appending them to the end of the sequence library. Searches were performed using the following parameters: 1.5 Da precursor ion mass tolerance, 0.4 Da fragment ion mass tolerance, 1 missed cleavage, alkylated Cys as a fixed modification and variable modifications of acetylated N-terminus and Lys, and oxidated Met. Searches were run in parallel on a 12 node, 24 CPU Linux cluster (Linux Networx, Bluffdale, UT).

False positive rates were determined using the method of Elias and co-workers^5^. Briefly, false positive rates were calculated by multiplying the number of false positive identifications (hits to the reversed sequences scoring below a given threshold) by 2 and dividing by the number of total identifications. Peptides identified below threshold, and also occurring as matches to the forward sequences, were not counted as false positives or true identifications. A curve was then generated by plotting E-value versus false positive rate and an E-value threshold corresponding to a 1% false positive rate was used as the cutoff in this analysis^6^. After generation of search data, the result files were parsed and loaded into a custom MySQL database for visualization and reporting using in-house software.

A total of 6,711 proteins were identified at a 1% false discovery rate (FDR) as determined by the target-decoy search method. Paired t-test p-values for each protein were calculated across all nine pairs of primary and recurrent ovarian cancer samples. The p-value distribution across all proteins is shown in Figure S2. The candidate proteins with high peptide counts and a p-value < 0.1 that exhibited more than 2-fold upregulation in at least 4 out of 9 recurrent cases compared to their primaries were selected and subsequently validated by qRT-PCR (see Table S2 for the expression data of the 6,711 proteins identified in this study). The largest number of cases showing more than 2-fold difference is 8 out of 9 pairs, and this was observed in 2 proteins, AIF1 and PLEK. Figure S3 shows the distribution of case pairs with more than 2-fold variation.

**References**

1. Wang, Y.; Rudnick, P. A.; Evans, E. L.; Zhuang, Z.; Li, J.; DeVoe, D. L.; Lee, C. S.; Balgley, B. M. *Anal. Chem.* **2005,** *77,* 6549-6556.
2. Wang, Y.; Balgley, B. M.; Lee, C. S. *Expert Rev. Proteomics* **2005,** *2,* 659-667.
3. Wang, Y.; Balgley, B. M.; Rudnick, P. A.; Evans, E. L.; DeVoe, D. L.; Lee, C. S. *J. Prot. Res.* **2005,** *4,* 36-42.
4. Geer, L. Y.; Markey, S. P.; Kowalak, J. A.; Wagner, L.; Xu, M.; Maynard, D. M.; Yang, X.; Shi, W.; Bryant, S. H. *J. Prot. Res.* **2004,** *3,* 958-964.
5. Elias, J. E.; Haas, W.; Faherty, B. K.; Gygi, S. P. *Nat. Methods* **2005,** *2,* 667-675.
6. Balgley, B. M.; Laudeman, T.; Yang, L.; Song, T.; Lee, C. S. Mol. Cell. Proteomics **2007,** *6,* 1599-1608.
