# Supplementary figures and images for: Oncoproteomic Analysis Reveals Co-Upregulation of RELA and STAT5 in Carboplatin Resistant Ovarian Carcinoma
Source: PLoS One. 2010 Jun 18;5(6):e11198. doi: 10.1371/journal.pone.0011198 (PMC2887843; doi:10.1371/journal.pone.0011198)

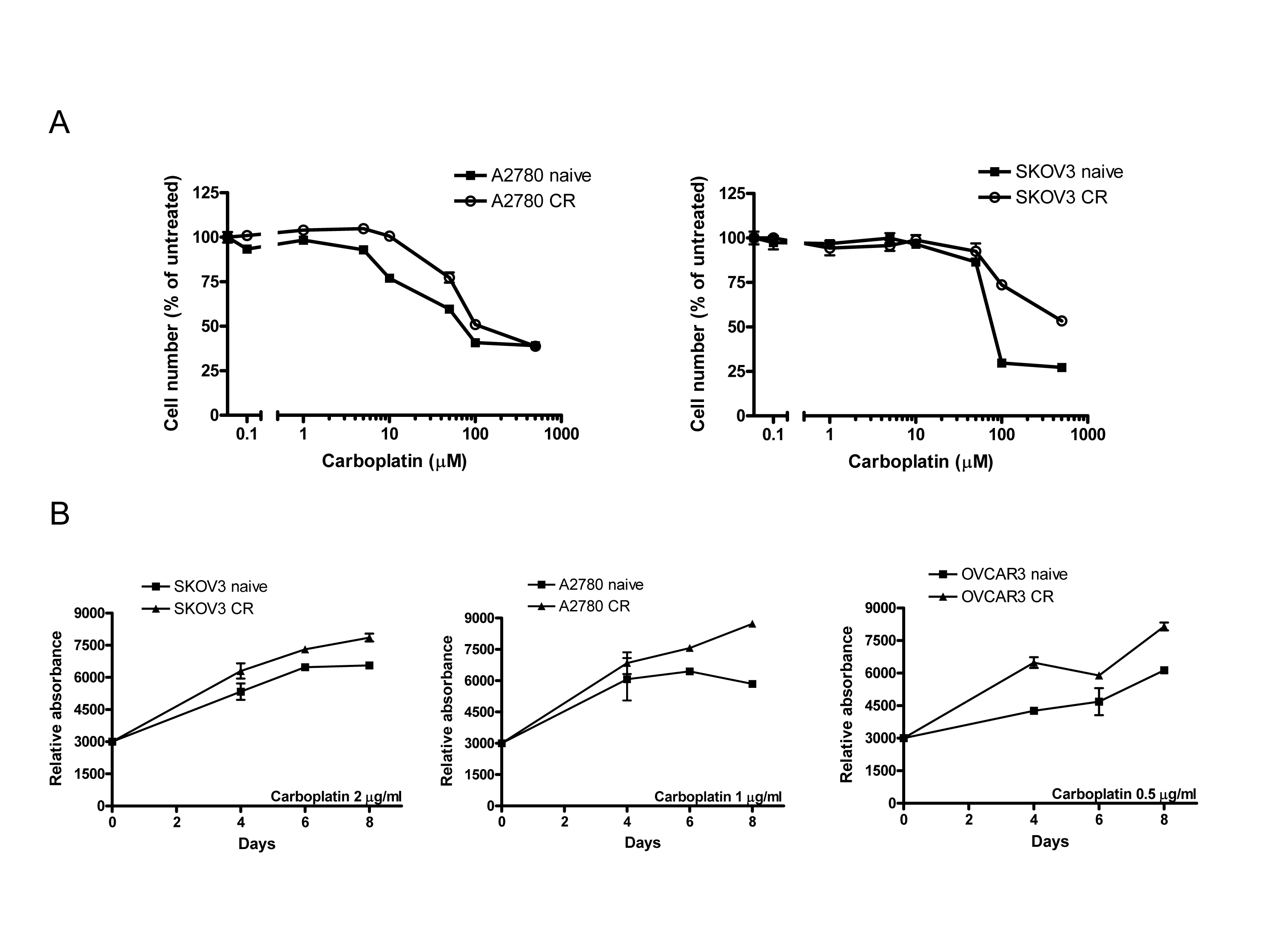

Supplement: Figure S1 — Carboplatin-resistant ovarian cancer cells A. Carboplatin IC50 of chemoresistant cells and their naïve counterparts [left panel: A2780; right panel: SKOV3]. B. Growth curve (cell viability assays) of carboplatin-resistant SKOV3, A2780, OVCAR3 cells and their naïve counterparts treated with 2 µg/ml, 1 µg/ml, and 0.5 µg/ml of carboplatin, respectively. (0.60 MB TIF) [file pone.0011198.s002.tif]

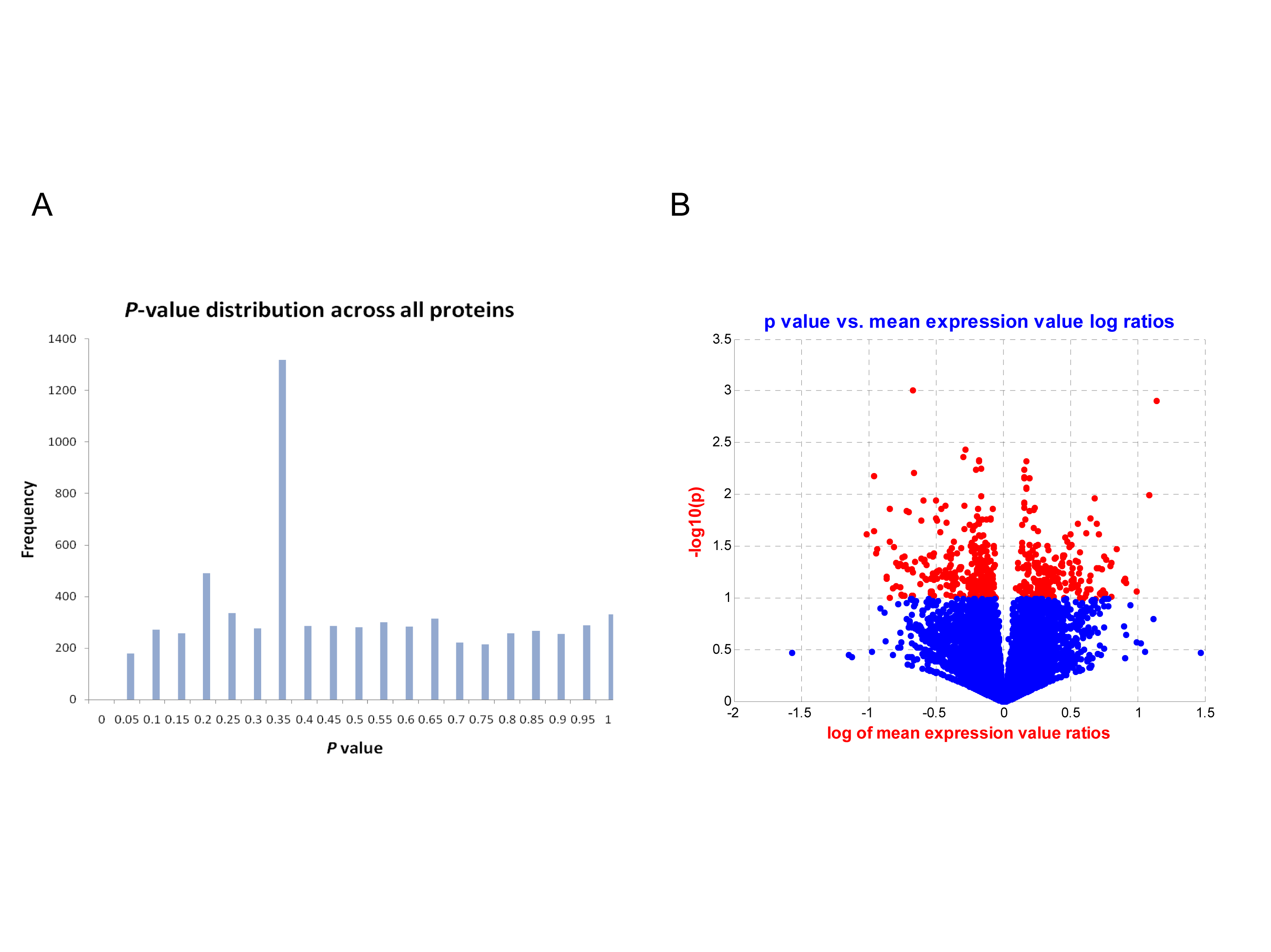

Supplement: Figure S2 — Paired t-test p-value distribution across the 6,711 proteins. A. A histogram plot of the p-value distribution across all proteins. B. A volcano plot of the p-value distribution across all proteins. (0.59 MB TIF) [file pone.0011198.s003.tif]

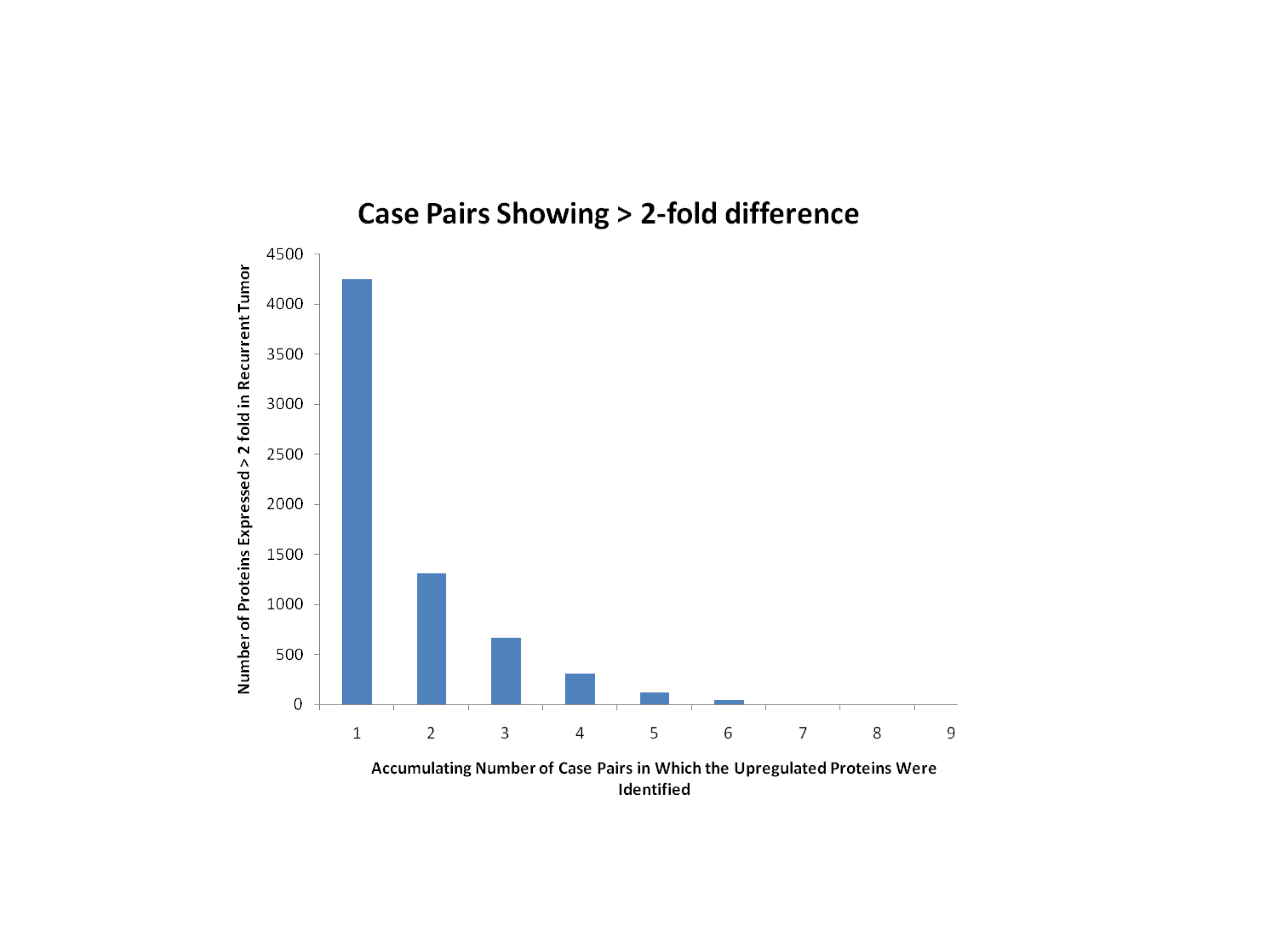

Supplement: Figure S3 — The distribution of case pairs showing more than 2-fold upregulation in recurrent compared to primary ovarian cancers. (0.53 MB TIF) [file pone.0011198.s004.tif]

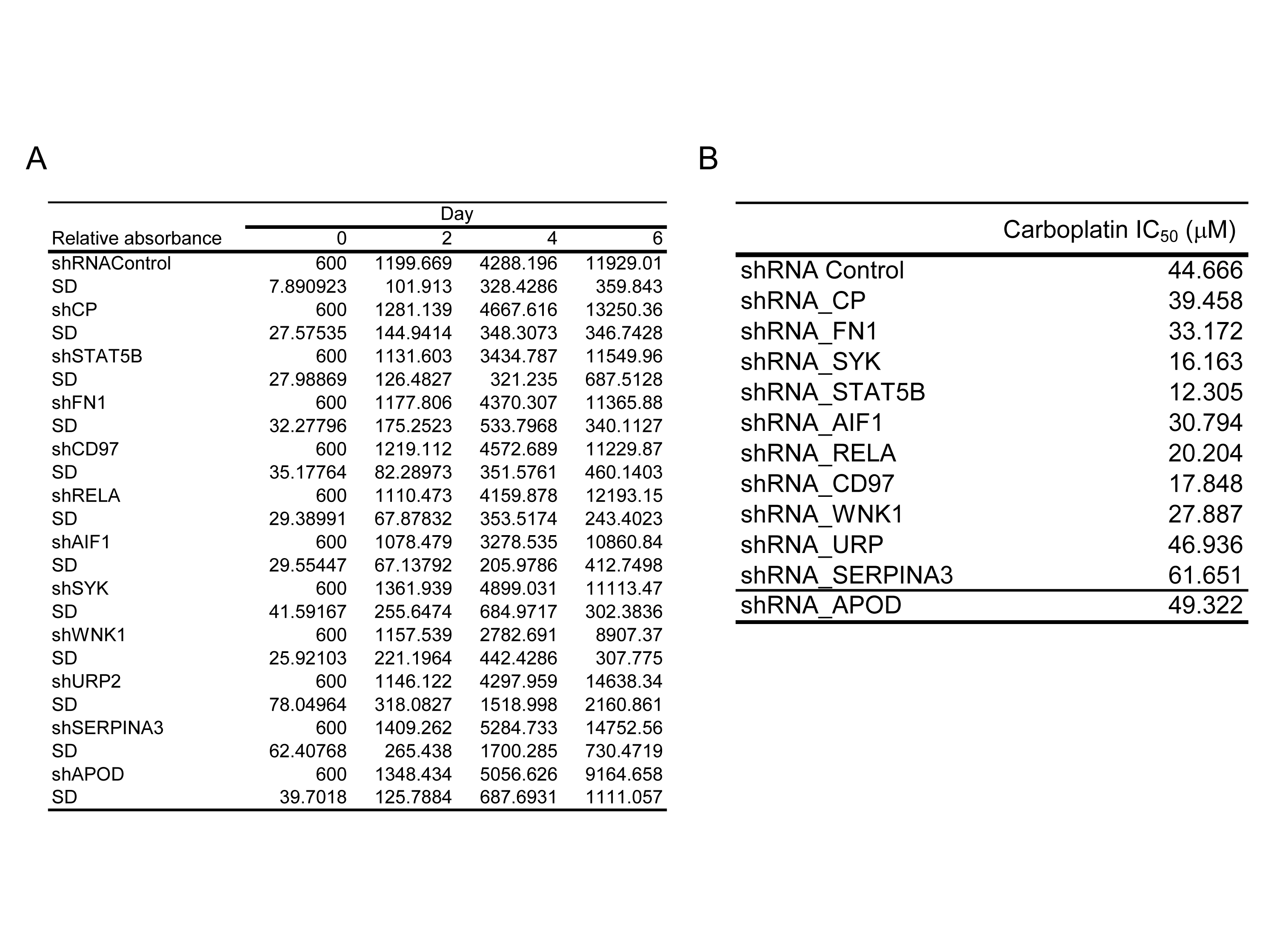

Supplement: Figure S4 — All shRNAs screening results using SKOV3-CR cells. A. Growth curve analysis of SKOV3-CR transfected with each shRNA. B. Carboplatin IC50 of each shRNA screened. (0.79 MB TIF) [file pone.0011198.s005.tif]
